# Colloidal quantum dot based solar cells: from materials to devices

**DOI:** 10.1186/s40580-017-0115-0

**Published:** 2017-08-07

**Authors:** Jung Hoon Song, Sohee Jeong

**Affiliations:** 1 Nano-Convergence Systems Research Division, Korea Institute of Machinery and Materials (KIMM), Daejeon, 34113 Republic of Korea; 2 Department of Nanomechatronics, University of Science and Technology (UST), Daejeon, 34113 Republic of Korea

**Keywords:** Colloidal quantum dots, Nanocrystals, Solar cells, Photovoltaics, Lead chalcogenides

## Abstract

Colloidal quantum dots (CQDs) have attracted attention as a next-generation of photovoltaics (PVs) capable of a tunable band gap and low-cost solution process. Understanding and controlling the surface of CQDs lead to the significant development in the performance of CQD PVs. Here we review recent progress in the realization of low-cost, efficient lead chalcogenide CQD PVs based on the surface investigation of CQDs. We focus on improving the electrical properties and air stability of the CQD achieved by material approaches and growing the power conversion efficiency (PCE) of the CQD PV obtained by structural approaches. Finally, we summarize the manners to improve the PCE of CQD PVs through optical design. The various issues mentioned in this review may provide insight into the commercialization of CQD PVs in the near future.

## Introduction

Colloidal quantum dots (CQDs) are chemically-prepared semiconductor nanocrystals, which have diameter is less than twice the Bohr radius describing the spatial extension of exciton (electron–hole pair) in semiconductors. The CQDs have attracted attention over the past decade due to a solution based synthetic methods [1], easily tunable optoelectronic properties [2], and superior processing capabilities for optoelectronic applications [3]. Specifically, lead chalcogenide CQDs are considered as a prominent material for a next-generation photovoltaics (PVs) [4] owing to their wide tunable bandgaps covering from visible to near-infrared wavelength regime arising from a large Bohr exciton radius and narrow bulk bandgap. Also, the excitons in lead chalcogenide CQDs can be easily separated into electrons and holes because of their high dielectric constant and extinction coefficient. More importantly, because their material properties can be easily controlled using electron density functional design, lead chalcogenide CQDs are expected to exhibit efficient utilization of low-energy and low-intensity photons and efficient collection of high-energy charges, which are difficult to achieve in conventional PVs [5]. In this article, we discuss the current state of high-efficiency CQD PV development.

## Size-dependent physical properties of CQDs

**Fig. 1:**
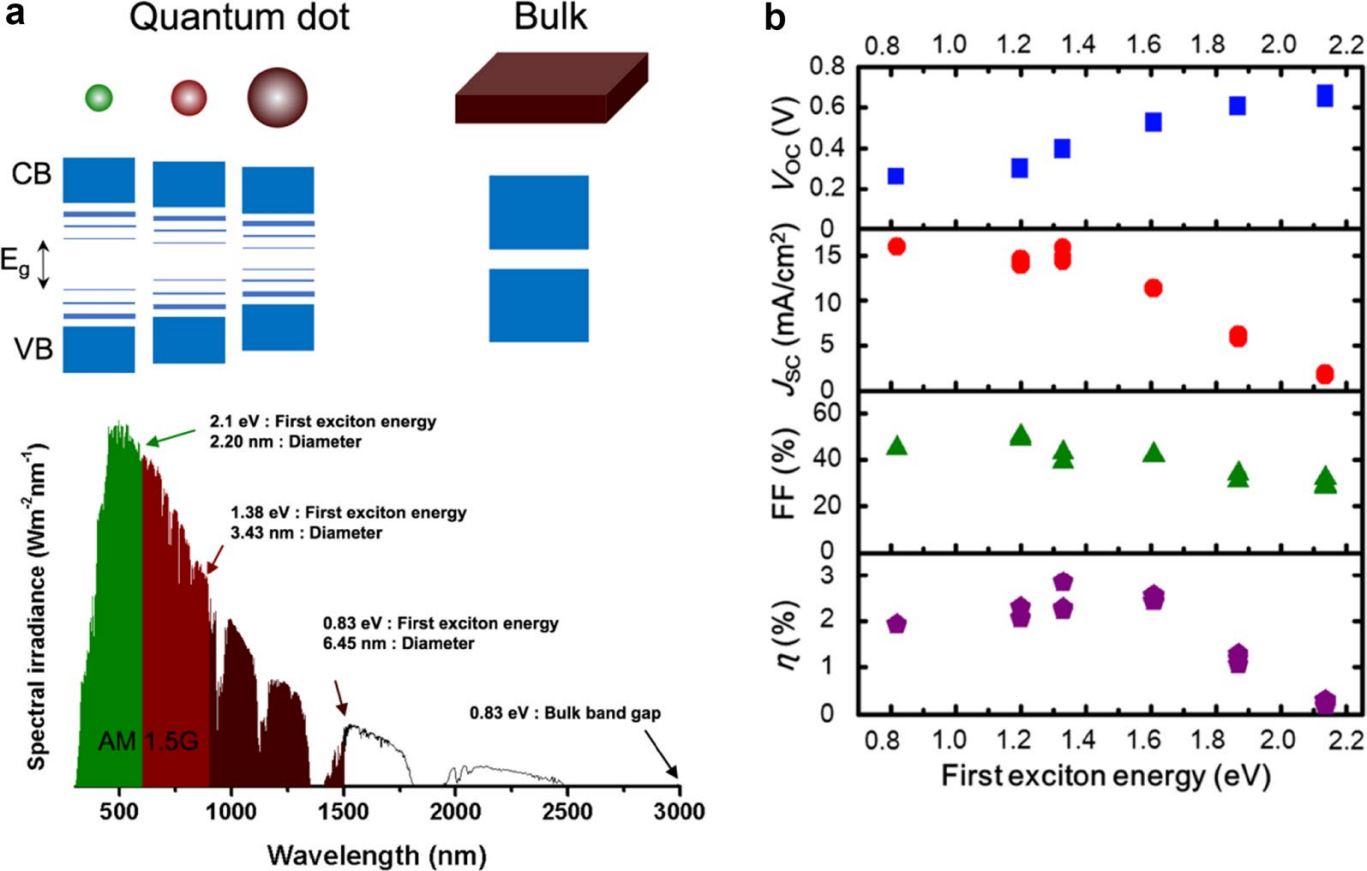
**a** AM 1.5G solar spectrum (from ASTM G173-03 reference spectra), band diagram, and first exciton energy of PbS CQDs with various diameters. **b** Characteristics of Schottky junction PbS CQD PVs as a function of the first exciton energy (reprinted with permission from ref. 6, Copyright 2013 American Physical Society)

When the size of a bulk semiconductor is reduced, discrete energy levels appear in the energy band owing to the quantum confinement effect, as shown in Fig. 1a. A CQD consists of a semiconductor core and surface ligands. In the case of a core, synthesis methods capable of controlling the size of binary or ternary compound semiconductors, such as II–VI (CdSe, CdS) compound semiconductors, III–V (InP, InAs), IV–VI (PbS, PbSe), and III–V (CuInS_2_, CuInSe_2_), have been developed and reported. As shown in Fig. 1a, the bandgap of IV–VI (PbS, PbSe) CQDs can be controlled to absorb light in the range of 600–3000 nm, which is suitable for solar cell materials. Additionally, most of the reported high-efficiency CQD PVs have been fabricated using IV–VI (PbS, PbSe) CQDs. Therefore, in this article, we mainly focus on the IV–VI (PbS, PbSe) CQDs. Typically, when the energy bandgap of a semiconductor decreases in a single-junction PV, the open-circuit voltage (V_OC_) decreases and the short-circuit current (J_SC_) increases by absorbing more light. Figure 1b shows that single-junction CQD PVs also exhibit the relationship between energy bandgaps and solar cell characteristics as described above [6]. In CQD solids, the hole mobility increases depending on the size of the CQDs [7], explained by the decrease in the total number of interparticle hops and the reduction of the coulombic charging energy of an individual particle.

## Surface modification and characteristics control

**Fig. 2:**
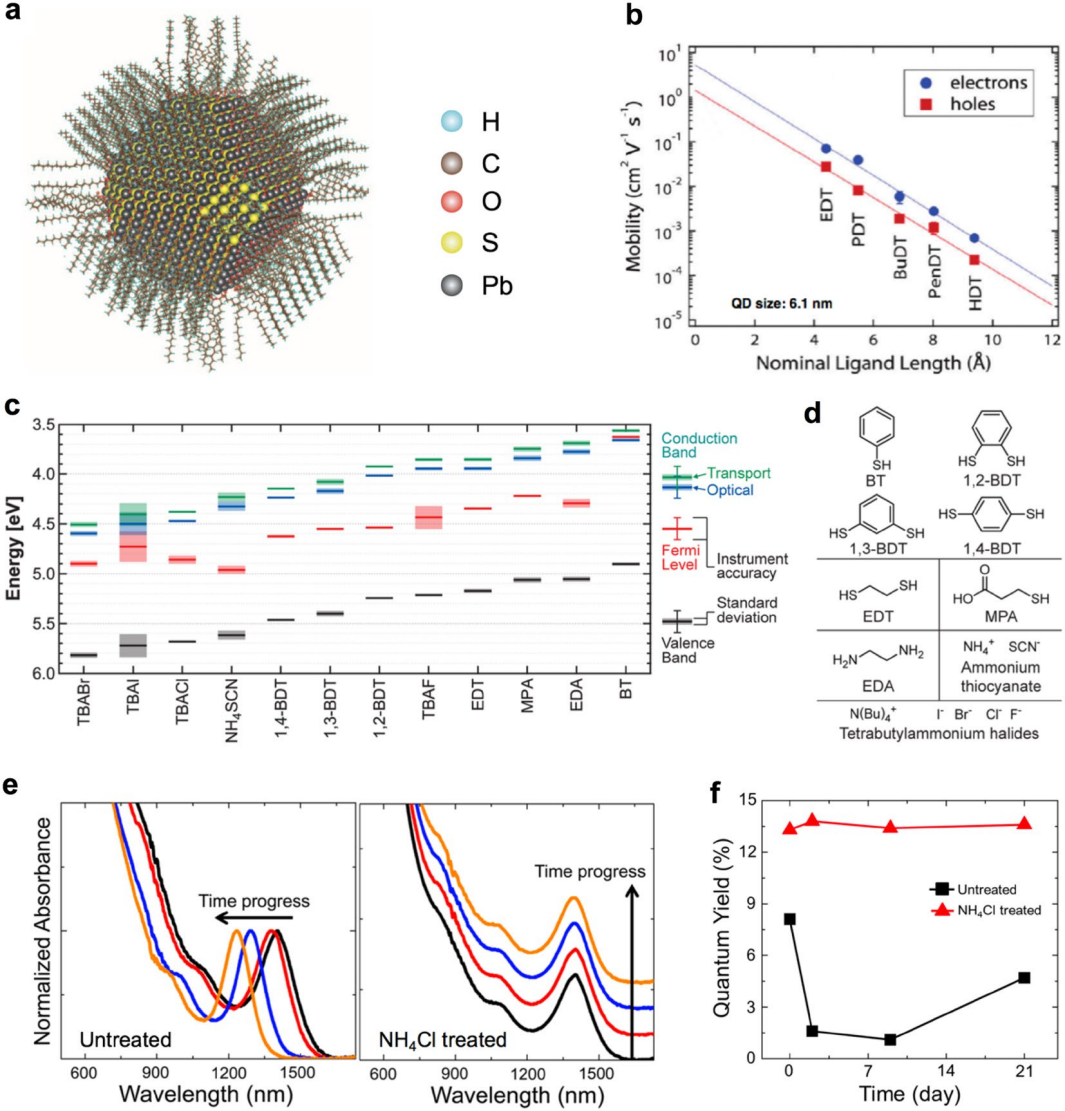
**a** Schematic diagram of a PbS CQD consisting of a semiconductor core and surface ligands (reprinted with permission from ref. 8, Copyright 2014 American Association for the Advancement of Science). **b** Charge carrier mobility of PbS CQD films with various surface ligand length (reprinted with permission from ref. 9, Copyright 2013 American Chemical Society). **c** Energy band position of PbS CQD films for **d** different surface ligands (reprinted with permission from ref. 10, Copyright 2013 American Chemical Society). Air stability analysis using **e** the absorbance spectrum and **f** quantum yield of PL as a function of storage time and its dependence on NH_4_Cl treatment on the surface of PbSe CQDs (reprinted with permission from ref. 11, Copyright 2014 American Chemical Society)

Nanomaterials such as CQDs generally have a high surface-area-to-volume ratio and the surfaces are prone to form dangling bonds, which causes defects. Especially, reducing surface defects is very important for obtaining high-efficiency CQD PVs. A CQD fabricated using wet-chemical synthesis consists of a semiconductor core and surface ligands, as shown in Fig. 2a [8]. The physical characteristics of the CQD can be controlled by the surface ligands owing to its high surface-area-to-volume ratio. Surface ligands generally have an amphiphilic structure consisting of a polar head group and a nonpolar aliphatic group. The rear portion of the ligand, which is composed of aliphatic groups, provides steric stabilization and dispersibility, which is the ability to dissolve in organic solvents. The head group typically contains amines, carboxylate, thiolate and phosphonate. These functional groups bind to the cationic metals on the CQD surfaces and produce nonstoichiometric CQDs, resulting in a doping effect. Typically, compound semiconductors exhibit p-type doping polarity under anion-rich conditions and n-type doping polarity under cation-rich conditions. Thus, doping can be controlled by using these ligands. When a CQD film is fabricated, the mobility of the charge carriers is determined by the length of ligands that passivate the surface of the CQDs. As shown in Fig. 2b, the mobility and coupling of the CQDs increase with the use of shorter-length ligands [9]. In addition, when surface ligands bind to the surface of CQDs, the electron distribution of the CQD surface and ligand changes, resulting in the formation of dipole moment on the CQD surface. The strength and direction of the surface dipole moment are determined by the surface ligands. Therefore, the position of the energy band is shifted by the surface dipole moment, as shown in Fig. 2c–d [10]. CQDs have many surface defects because of their high surface-area-to-volume ratio. Therefore, the surface ligands play an important role in reducing these surface defects. Figure 2e–f show that additional passivation using halide atoms reduces the surface defects of CQDs and increases photoluminescence (PL) [11, 12]. Furthermore, as passivation prevents the oxidation of CQDs, their air stability can be improved [11, 12]. Consequently, the surface ligands of CQDs play an important role in controlling the dispersibility, air stability, and electrical properties (such as doping, mobility, electronic structure, and surface defects). Therefore, understanding the dependence of CQD characteristics on surface ligands is essential for obtaining high-efficiency CQD PVs.

## Enhancement of power conversion efficiency through solar cell structure design

**Fig. 3:**
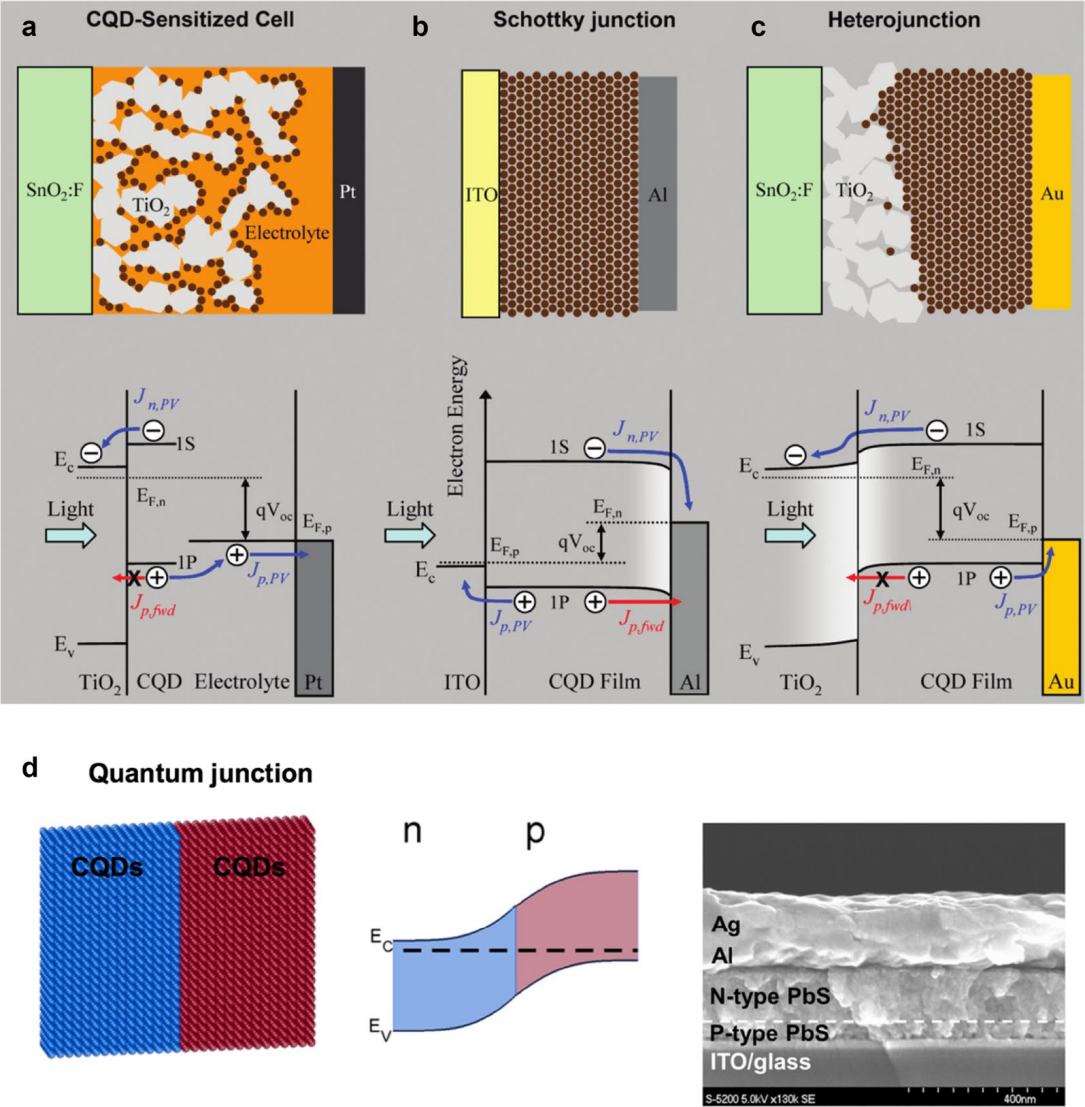
Schematic of photovoltaic architectures and flat-band diagrams at V_OC_ of **a** CQD-sensitized solar cell, **b** Schottky junction solar cell, and **c** heterojunction solar cell (reprinted with permission from ref. [13], Copyright 2010 American Chemical Society). **d** Schematic of CQD homojunction solar cell, energy band diagram, and cross-sectional image of scanning electron microscope image (reprinted with permission from ref. [19], Copyright 2012 American Chemical Society)

The power conversion efficiency (PCE) of CQD PVs has increased very rapidly since national renewable energy laboratory (NREL) certification began in 2010 and the highest PCE in the NREL chart is currently 13.4%. In the early stages of the development of CQD PVs, the PCE was increased in accordance with the structural changes of the devices. However, since 2012, the development of technologies that control the surface of CQDs has resulted in dramatic improvements of the performance of CQD PVs. In Sect. 4, we will describe the structural development of CQD PVs. Initially, CQDs with high extinction coefficients were used instead of dyes in dye-sensitized solar cells. In this case, the CQD absorbs light to form excitons, and electrons and holes separated from the excitons are generally transferred through TiO_2_ and the electrolyte, respectively (Fig. 3a) [13]. Consequently, the electric characteristics of the CQDs have relatively low influence on the device in the dye-sensitized solar cell. However, the CQDs with uncontrolled electrical properties can be formed as a monolayer on TiO_2_ to absorb only a small amount of light. Therefore, mesoporous TiO_2_ is commonly used to overcome this problem. In recent years, new CQDs without heavy metals (such as Zn–Cu–In–Se CQDs) have been employed to produce a dye-sensitized solar cell structure that exhibits a relatively high PCE [14–16].

Currently, most of the high-efficiency CQD PVs use a thin film solar cell structure. For the PbS CQD solar cells, the excitons generated by light are easily separated by the internal field of the diode due to their high dielectric constant, and the separated electrons and holes move in the CQD thin film. Therefore, their electronic properties itself largely influence on the CQD solar cells. Such electronic properties can be controlled by chemically surface treatment. Initially, thin-film CQD PVs used a Schottky diode structure with a metal (Fig. 3b) [13, 17]. However, the QD film thickness cannot be increased, originated by the small built-in potential and narrow space charge region (SCR) in such Schottky junction CQD PVs. In addition, their charge carrier diffusion length is very short (several tens of nanometers) due to many surface trap states. For the high-efficiency CQD PVs, the SCR should be enlarged. To overcome this problem, heterojunction diodes have been fabricated using oxide semiconductors with n-type doping polarity such as ZnO and TiO_2_ (Fig. 3c) [13, 18].

Finally, a homojunction CQD PV with CQDs of n-type and p-type doping polarities has been successfully developed by stoichiometry control using surface ligands (Fig. 3d). The structure of the homojunction shows a lower efficiency than heterojunction structures. In a heterojunction using oxide semiconductors with relatively large energy bandgaps, such as ZnO and TiO_2_, the electrons move easily at the interface between the CQD and the oxide semiconductor; however, the holes cannot move across the interface and the recombination of electrons and holes decreases. Therefore, the homojunction CQD PVs show lower PCE than heterojunction ones [19].

## Enhancement of power conversion efficiency through surface modification

**Fig. 4:**
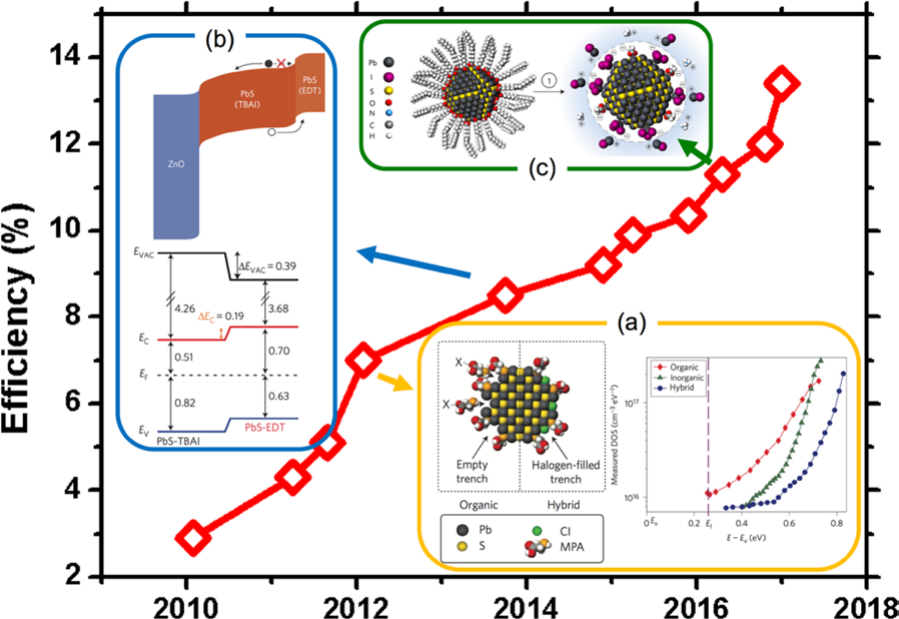
NREL efficiency chart of CQD PVs. *a* Schematic of a CQD treated with an MPA ligand and a chloride (inorganic) ligand; density of states in the energy bandgap obtained from a transient photo-voltage measurement (reprinted with permission from ref. 21, Copyright 2012 Macmillan Publishers Ltd.). *b* Band diagram of a CQD PV based on electron blocking with EDT-exchanged CQDs; energy band positions for various ligands obtained from ultraviolet photoelectron spectroscopy analysis (reprinted with permission from ref. 22, Copyright 2014 Macmillan Publishers Ltd.). *c* Schematic of CQD and CQD ink (reprinted with permission from ref. 25, Copyright 2016 Macmillan Publishers Ltd.)

The surface modification of a CQD is an important factor that determines not only the characteristics of the CQD but also the characteristics of the resulting PV device. Figure 4 shows the efficiencies of CQD PVs, as reported by the NREL chart. As shown in the efficiency chart, there are significant developments of the CQD PVs efficiencies over the last 7 years as understanding and controlling the surface of CQDs. In the case of CQDs fabricated by wet-chemical synthesis, the surface is passivated by long organic ligands to reduce internal defects and improve size uniformity due to high synthesis temperatures. To fabricate a thin-film CQD PV, long organic ligands must be replaced with short ligands to improve carrier mobility. When forming a conductive CQD thin film, a layer-by-layer (LBL) process is used to repeatedly perform the deposition of the CQD film and the ligand exchange process to reduce cracks. After the synthesis, PbS CQDs consist of Pb-rich nonstoichiometric (111) surfaces passivated by ligands and stoichiometric (100) surfaces without ligands [20]. The long organic ligands on the non-stoichiometric (111) surface are replaced by mercaptopropionic acid (MPA) ligands containing sulfur (anion), leading to the p-type behavior in CQDs. Thus a heterojunction CQD PV with the p-type of CQDs and n-type of TiO_2_ or ZnO can be fabricated [21]. As mentioned in Sect. 4, the CQDs have many surface defects and a very short diffusion length; and thus charge carriers are mainly driven by the drift in the electric field inside the SCR. Therefore, to achieve high-efficiency CQD PVs, the CQD active layer should increase without decreasing the extraction efficiency of photo-induced carriers through improving the carrier diffusion length. The (100) surface has no surface ligands and is easily oxidized to generate defects. When producing CQD PVs with halide-treated CQDs (Fig. 2e–f), both the air stability can be improved and the efficiency can be increased by improving carrier diffusion length due to reduction of surface defects (Fig. 4a) [21].

In addition, the electron blocking layer (EBL) at the interface between the CQD thin film and the metal electrode is used in the same principle to prevent recombination by blocking the hole transport at the interface. In this case, EBL prevents the movement of electrons to the metal electrode through the energy band shift of ethanedithiol (EDT)-treated CQD film [22]. Therefore, high-efficiency CQD PVs can be achieved by using an EBL to reduce electron recombination. In this structure, the number of surface defects and the type of ligands vary depending on the acidity of the protonic solvent used in the ligand exchange process. An efficiency higher than 10% has been reported for CQD PVs produced by changing the electrical properties of CQD films with various protic solvents in the ligand exchange process (Fig. 4b) [23].

The fabrication of CQD PVs is based on the LBL process by employing the above ligand exchange process. This fabrication procedure is time-consuming and the electrical properties of the CQD film are modified by the environment during the ligand exchange process. Therefore, the LBL process reduces the reproducibility of CQD PVs and inhibits their commercialization. To solve this problem, the surface of PbS CQDs has been completely modified with ammonium iodide (NH_4_I), which has the closest affinity with Pb, to allow the CQDs to be dissolved in *N*, *N*-dimethylformamide [24]. Using a technique similar to the above method and considering the dependence of the energy band shift on the ligand, CQDs with a PCE of 11.3% have been obtained (Fig. 4c) [25]. Ligand exchange performed in the liquid phase is more complete than solid ligand exchange because it increases the diffusion length by suppressing surface defects, resulting in increased thickness of the CQD film without reducing the efficiency.

## Enhancement of power conversion efficiency using optical design

**Fig. 5:**
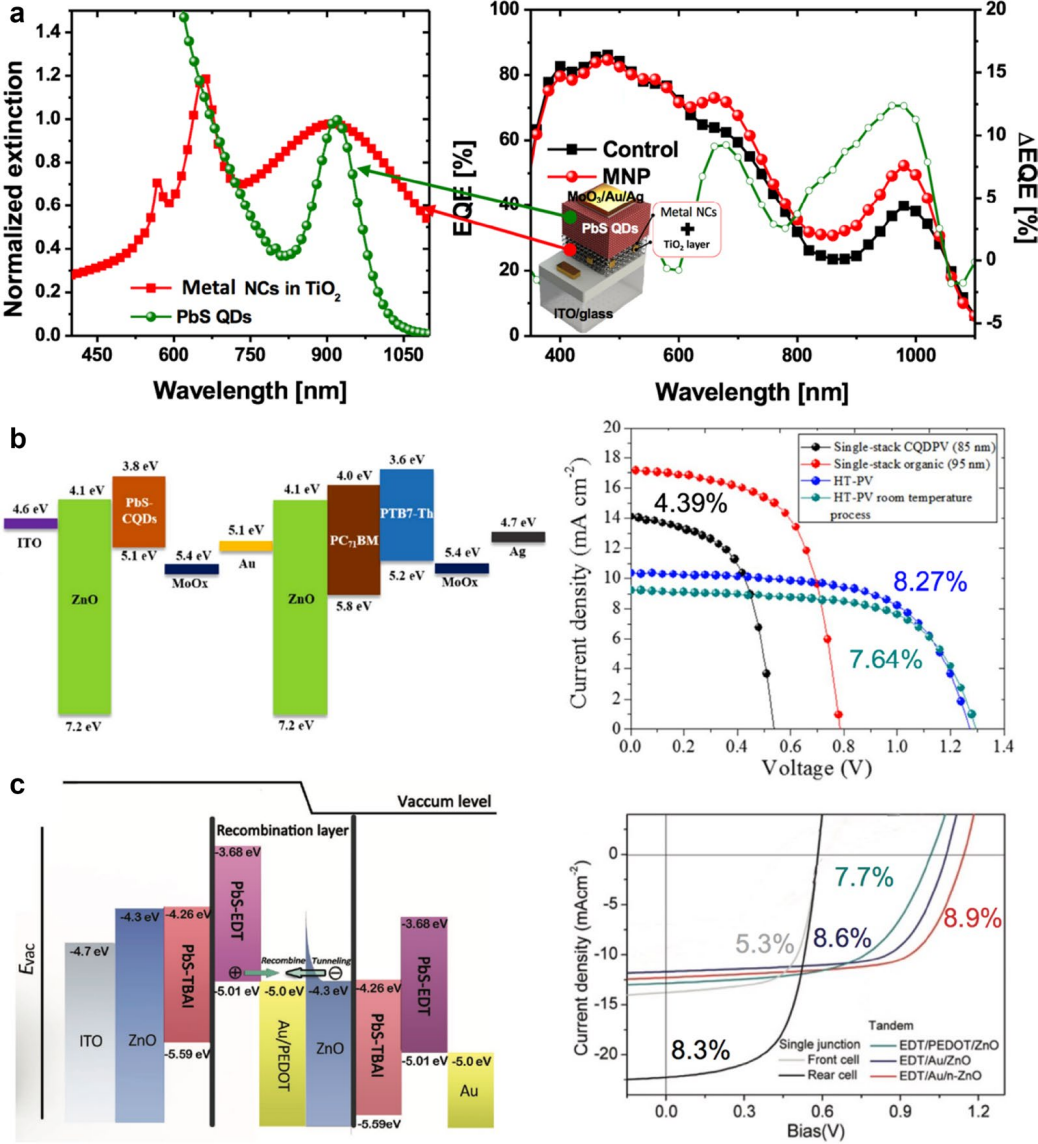
**a** Absorption spectra of metal nanoparticles in TiO_2_ and PbS CQDs; EQE spectra with and without metal nanoparticles (reprinted with permission from ref. 26, Copyright 2015 Wiley–VCH Verlag GmbH & Co. KGaA). **b** Energy band diagram and J–V curves of multi-junction PVs using an organic PV and a CQD PV (reprinted with permission from ref. 27, Copyright 2016 Elsevier B.V.). **c** Energy band diagram and J–V curves of multi-junction PVs using CQD PVs (reprinted with permission from ref. 28, Copyright 2017 Wiley–VCH Verlag GmbH & Co. KGaA)

In CQDs, the quantum confinement effect separates the levels of the energy band of bulk semiconductors, resulting in an increase in the energy bandgap and a discrete energy level, as shown in Fig. 1a. This discrete energy level decreases the density of state and reduces the amount of absorbed light around the energy bandgap. Therefore, observations of the external quantum efficiency (EQE) spectrum of a CQD PV show a markedly decreased efficiency near the energy bandgap. This is a unique characteristic of the CQD. To solve this problem, surface plasmons of metal nanoparticles have been used to shift the spectrum of the incident sunlight to the region in which CQDs can absorb large amounts of light, thereby improving the PCE (Fig. 5a) [26].

Multiple junctions using CQDs improve the PCE because they provide easier control of the light absorption region of the CQDs, thus adjusting the energy bandgap. Although relatively few studies have been conducted on multi-junction CQD PVs, they have shown a possibility of maximized efficiency. The LBL process is performed to fabricate the conductive CQD film. In this case, because an organic ligand has a specific acidity and LBL process is performed using a polar solvent and a nonpolar solvent alternately, the pre-formed front sub-cell and intermediate recombination layer (RL) is damaged and the efficiency is lowered. Therefore, as shown in Fig. 5b, a CQD PV with a smaller bandgap than that of the organic PV is placed in the front sub-cell, thereby minimizing the reduction in efficiency [27]. In addition, intermediate RLs have been developed that can induce sufficient electron and hole recombination without damage by using the solution process during the formation of the rear sub-cells to minimize the efficiency reduction (Fig. 5c) [28]. However, the current efficiency of multi-junction CQD PVs is lower than that of single-junction CQD PVs; therefore, further research is required to address these problems. One possible solution is to minimize the damage of the front sub-cell by using CQDs in which the ligand is exchanged in the liquid phase mentioned in the previous section.

## Conclusions

CQDs have been attracting much attention because they can absorb light above their energy bandgap with a high extinction coefficient and can be processed by using a solution process. Significant progress has been achieved in the development of CQD PVs by understanding their surface characteristics and using surface modification to obtain superior characteristics that distinguish them from general bulk semiconductors. In the future, we aim to address the various issues presented in this article and to commercialize CQD PVs along with the commercialization of CQD display fields.
